# Neuromodulation and Synaptic Plasticity for the Control of Fast Periodic Movement: Energy Efficiency in Coupled Compliant Joints via PCA

**DOI:** 10.3389/fnbot.2016.00002

**Published:** 2016-03-08

**Authors:** Philipp Stratmann, Dominic Lakatos, Alin Albu-Schäffer

**Affiliations:** ^1^Department of Informatics, Sensor Based Robotic Systems and Intelligent Assistance Systems, Technische Universität MünchenGarching, Germany; ^2^Institute of Robotics and Mechatronics, German Aerospace CenterWeßling, Germany

**Keywords:** movement generation, compliant actuators, control theory, spike-timing-dependent plasticity, neuromodulation, principal component analysis

## Abstract

There are multiple indications that the nervous system of animals tunes muscle output to exploit natural dynamics of the elastic locomotor system and the environment. This is an advantageous strategy especially in fast periodic movements, since the elastic elements store energy and increase energy efficiency and movement speed. Experimental evidence suggests that coordination among joints involves proprioceptive input and neuromodulatory influence originating in the brain stem. However, the neural strategies underlying the coordination of fast periodic movements remain poorly understood. Based on robotics control theory, we suggest that the nervous system implements a mechanism to accomplish coordination between joints by a linear coordinate transformation from the multi-dimensional space representing proprioceptive input at the joint level into a one-dimensional controller space. In this one-dimensional subspace, the movements of a whole limb can be driven by a single oscillating unit as simple as a reflex interneuron. The output of the oscillating unit is transformed back to joint space via the same transformation. The transformation weights correspond to the dominant principal component of the movement. In this study, we propose a biologically plausible neural network to exemplify that the central nervous system (CNS) may encode our controller design. Using theoretical considerations and computer simulations, we demonstrate that spike-timing-dependent plasticity (STDP) for the input mapping and serotonergic neuromodulation for the output mapping can extract the dominant principal component of sensory signals. Our simulations show that our network can reliably control mechanical systems of different complexity and increase the energy efficiency of ongoing cyclic movements. The proposed network is simple and consistent with previous biologic experiments. Thus, our controller could serve as a candidate to describe the neural control of fast, energy-efficient, periodic movements involving multiple coupled joints.

## 1. Introduction

During fast periodic motions, such as jumping or drumming, animals exploit the natural dynamics of their elastic locomotor systems to achieve high velocity in an energy-efficient manner (Bar-Cohen, 2011, p. 514). Their central nervous systems (CNSs) are able to quickly adjust the control of periodic movements that involve several joints to face changes of their environment or intrinsic body properties (Hatsopoulos and Warren, 1996; Zondervan et al., 2014). The underlying control problem is highly complex, as the locomotor systems have multiple joints that have non-linear compliances and are dynamically coupled. For a controller algorithm to replicate the CNS's locomotion control, it must be able to induce stable movement and quickly tune it to high energy efficiency under varying mechanical conditions, while being consistent with biological experiments.

Fast, or explosive, movements such as jumping are typically compound movements that involve synchronous trajectories of several joints in a single or several limbs (Freund and Büdingen, 1978; Morasso, 1981). The synchronicity enables high maximum force and thereby allows to take advantage of elastic dynamics. This can increase the resulting energy efficiency and thereby movement speed. Energy efficiency implies that for constant energy input a controller increases the energy within a mechanical system, as e.g., represented by an increased jump height (cf. Section 4.7.3). In systems with one degree of freedom, maximum energy efficiency implies correct timing of the controller output. In natural explosive movements involving several joints, it also requires the adjustment of the relative amplitude of motor signals at different joints. For the remainder of this article, the latter mechanism shall be denoted as *intra-limb coordination*.

In neuroscience, both theoretical and experimental studies have described neural mechanisms that can induce stable movements in an elastic locomotor system via central pattern generators (CPGs) or reflex arcs (cf. Buschmann et al., 2015 for a review). Theoretical research has extensively analyzed the question on how compliant systems can be tuned to yield energy-efficient movements on artificial models with a single joint (Brambilla et al., 2006; Righetti et al., 2006; Pelc et al., 2008; Barikhan et al., 2014; Huang et al., 2014). Studies considering multiple joints showed that frequency adjustment can be achieved by multiple coupled CPGs, one for each joint involved, that are entrained to proprioceptive input. Multiple CPGs are especially beneficial in non-synchronous movements of the joints, where phase-tuning between different joints is required and where different joints in a limb could execute functionally different tasks, such as forward/backward movement vs. elevation/depression in insect gaits (Nachstedt et al., 2012; Xiong et al., 2015). Buchli and Ijspeert (2008) demonstrate that multiple coupled CPGs, one for each actuated joint, can also be used to find the resonance frequency of fast compound periodic movements. However, the use of multiple CPGs neglects the described synchronicity in joint trajectories. Furthermore, tuning for higher energy efficiency also requires intra-limb tuning, i.e., to adjust the relative amplitude of motor signals at different joints.

Previous experimental research has considered both frequency and intra-limb tuning. Measurements on decerebrate cats demonstrated that signals from individual group I nerves converge in spinal pathways to entrain the frequency of all muscles involved (Whelan et al., 1995a; Hiebert et al., 1996). The efficacy of individual nerves to cause entrainment is dependent on their activity. The influence of a silenced nerve decreases with time, whereas an increased influence is found for nerves originating from muscles that assist in the same movement as the silenced one (Whelan et al., 1995b). Intra-limb coordination of explosive movements was found to be controlled by circuits in the brain stem and cerebellum (MacKay-Lyons, 2002; Shemmell et al., 2009). Furthermore, Animal studies found a disruption of intra-limb coordination after administration of a serotonin-antagonist (Pearlstein et al., 2005; Harris-Warrick, 2011). Serotonin (5-HT) metabotropically increases the excitability of motoneurons (Heckmann et al., 2005; Heckman et al., 2008; Perrier et al., 2013). It is released into the spinal cord by the raphe nucleus obscurus, pallidus and medianus (Jacobs et al., 2002), which reside in the brain stem. Since they receive proprioceptive input (Springfield and Moolenaar, 1983), the raphe neurons may be part of a motor feedback loop. The resulting absolute strength of motor signals during ballistic periodic movements can largely exceed the signal during maximum voluntary contractions (Dietz et al., 1979). Despite these experimental findings, neural pathways underlying the control of stable and energy-efficient explosive movements are poorly understood (Taube et al., 2012). In summary, current knowledge about the algorithm that the CNS encodes to tune ballistic periodic movements does not explain how the CNS maintains stable movement while tuning the frequency and inter-joint coordination to high energy efficiency. A physically motivated theoretical control approach would allow to link the experimental knowledge into a comprehensive framework.

Roboticists increasingly mimic the non-linear compliances of muscles and tendons in joints of mechanical robotic systems such as *BigDog* by *Boston Dynamics* (Raibert et al., 2008) or the *Hand Arm System* from the *German Aerospace Center* (*DLR*; Grebenstein et al., 2011). The control approaches developed by robot designers for controlling these bio-inspired robots can be a valuable source of hypotheses for neuroscientists. Several control algorithms have been suggested to induce stable and energy-efficient limit-cycle movements in compliant hybrid systems. However, their characteristics disqualify most designs as hypothesis for neural movement control. Van-der-Pol oscillators (Stramigioli and van Dijk, 2008) artificially damp systems and thereby reduce the energy efficiency of the movement. Poincaré-map based algorithms (Sreenath et al., 2010) cannot adequately adjust to different environments due to their dependence on a prior model and a fixed set of considered initial conditions. The same point argues against optimal-control algorithms, which additionally require numerical approaches and thus high computational power for multiple joints (Braun et al., 2011).

In this study, we propose an algorithm that was purely derived by engineering considerations on the control of biomechanically inspired robotic systems, to describe how the CNS may tune ballistic periodic movements to energy efficiency. We have previously shown that under specific intrinsic damping properties of muscles, tendons, and joints, the control of fast periodic movements can be reduced to exciting the local linear approximation of the non-linear mode of the system (Lakatos and Albu-Schäffer, 2014a; Lakatos et al., 2014). The corresponding algorithm linearly transforms sensory input from the multi-dimensional joint space into a one-dimensional controller space. The input entrains a driving unit, and the driving motor output is reversely transformed into the joint space. Multiplicative transformation weights are recurrently adapted and a driving unit as simple as a single reflex interneuron can adjust movements to unknown oscillatory patterns within few step cycles (Lakatos et al., 2013a,b).

Our algorithm does not share the adverse characteristics with the previous robotic control approaches mentioned above. It requires no prior model but needs only sensory information about joint deflections or forces. Additionally, the algorithm performs only linear calculations. This agrees with the recent findings from calculations performed by spinal interneurons (Spanne et al., 2014). In our previous work, we analytically proved stability of controlled mechanical systems with a single degree of freedom (Lakatos and Albu-Schäffer, 2014b). We numerically demonstrated stability in simulations for a controlled quadruped with 12 hinge joints (Lakatos and Albu-Schäffer, 2014a) and in a real robotic platform with four joints (Lakatos et al., 2013b).

For the remainder of this paper, we propose an exemplary neural network implementation of this algorithm in Section 2. By theoretical considerations and simulations of this network in Sections 2.2 and 2.3, respectively, we justify that the algorithm proposed by Lakatos et al. (2013b) may be implemented by the CNS to control fast periodic movements that involve several synchronously moving joints. At the input stage, we suggest that proprioceptive input converges from all muscles involved in a movement onto a single interneuron. Synaptic weights can align with the appropriate linear transformation weights under the influence of spike-timing-dependent plasticity (STDP). At the output stage, we show that serotonergic amplification of motoneuron output can produce the reverse transformation via the described motor feedback of medullary raphe nuclei. Our simulations substantiate that the proposed network can induce highly energy-efficient, stable, periodic movements in mechanical systems of different complexity. While we demonstrate in Section 3.2.2 that our neural sub-networks are consistent with previous experiments, we emphasize that our general controller design may be implemented by alternative circuits. Therefore, we discuss general mathematical requirements set by the controller and provide experimentalists with a checklist of necessary characteristics of a neural implementation in Section 3.3.

Our proposed transformation provides a functional unit that drives several joints with a sensory entrainment signal. The reverse transformation applied to the driving signal leads to correct intra-limb coordination. We argue in the discussion that the driving unit itself can be a pool of reflex interneurons, a CPG or a combination of both.

## 2. Results

**Figure 1:**
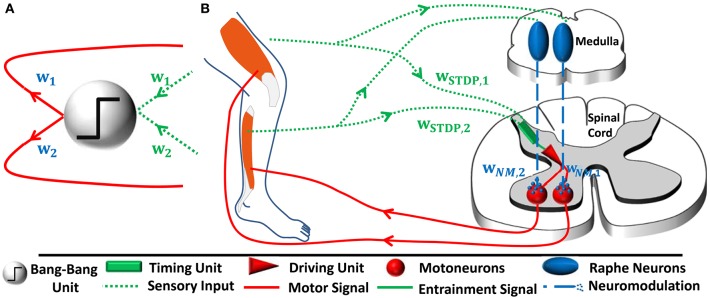
**(A)** In the mathematical controller design as proposed by Lakatos et al. (2013b), sensory input from each joint *i* is transformed into a one-dimensional coordinate space. For this purpose, the sensory inputs are multiplied by weights *w*_*i*_. The input entrains a thresholding bang-bang unit, which produces a motor signal. The driving signal is transformed back into the original joint space via the same respective weights. The output transformation accomplishes correct relative force weighting of the individual joints (Figure based on Lakatos and Albu-Schäffer, 2014a). **(B)** In our hypothetical neural controller implementation, sensory input from the joints also converges. The synaptic weights *w*_*STDP, i*_ change according to spike-timing-dependent plasticity. We emphasize that the bang-bang controller can be separated into two functionally distinct unit: A timing unit entrains a driving unit, which sends motor signals to muscles innervating all joints involved in a given periodic movement. While functionally distinct, these units do not have to be spatially separated in general. In our model, a single pool of reflex interneurons represents both units and outputs correctly timed motor signals. A parallel, joint-specific, sensory feedback pathway via raphe nuclei releases serotonin into motorpools. This amplifies the common motor output by *w*_*NM, i*_ and increases the relative strength of muscles that are more involved in the movement.

Following an overview on the controller introduced by Lakatos et al. (2013b) and illustrated in Figure 1A (cf. Section 2.1.1), we present models of two neural sub-networks that we propose based on previous animal experiments (cf. Sections 2.2.1 and 2.2.2, Figure 1B). We theoretically demonstrate that the network performs the proposed coordinate transformations.

In Section 2.3, we simulate our sub-networks in closed-loop simulations to verify that they can reliably excite stable, energy-efficient periodic movement. Detailed methodological descriptions of the simulations can be found in Section 4.

### 2.1. Controller theory

#### 2.1.1. Basic controller concept

We consider fast periodic movements with high synchronicity in the joint trajectories. The mathematical controller receives sensory information describing the observed movement, represented by the deflections φ_*i*_(*t*) of joints *i* from their respective zero position. Using the joint-specific weight vector with entries *w*_*i*_, sensory signals are linearly combined to obtain a single controller coordinate

(1) $$\begin{array}{l} \varphi_{z}\left(t\right)=w^{T}\left(t\right)\varphi \left(t\right). \end{array}$$

All motor units receive the same timing signal *f*_*z*_(*t*) that initiates force production when φ_*z*_ crosses a threshold,

(2) $$\begin{array}{l} f_{z}(t)=\{\begin{array}{ll} {\hat{c}}_{f} & \text{if }\varphi_{z}(t)>c_{\epsilon }\\ 0 & \text{otherwise}, \end{array} \end{array}$$

where ĉ_*f*_ and *c*_ϵ_ are positive constants. Equation (2) functionally describes both the timing and driving unit as illustrated in Figure 1B. It is transformed back into a change of force, *f*_*i*_(*t*), in the individual joints *i* by multiplication with the same weight vector ***w***,

(3) $$\begin{array}{l} \text{f}\left(t\right)=\text{w}\left(t\right)f_{z}\left(t\right). \end{array}$$

Exerting these forces on the joints induces stable energy-efficient movements due to correct timing and relative force amplitude (Lakatos et al., 2013b,a).

#### 2.1.2. Transformation weights

In this study, we focus on the adaptation of the weight vector ***w***. It is recursively updated and supposed to converge toward the dominant principal component of the data covariance matrix of the movement, which we denote the principal oscillation mode of the system. This can be achieved using

(4) $$\begin{array}{l} \frac{d}{dt}\text{w}\left(t\right)=\gamma \left[{\text{w}}^{T}\left(t\right)\varphi \left(t\right)\right]\left(\varphi \left(t\right)-\left[{\text{w}}^{T}\left(t\right)\varphi \left(t\right)\right]\text{w}\left(t\right)\right), \end{array}$$

where γ≪1 (Oja, 1992). The formula keeps weights bounded and generally increases the relative magnitude of weights for joints that are heavily involved in a movement.

It is assume that neuroscientific quantities representing weights *w*_*i*_ and sensory input φ_*i*_ are positive. We prove in the Presentation 1 that under this assumption the simplified formula

(5) $$\begin{array}{l} \frac{d}{dt}\text{w}\left(t\right)=c_{w}\varphi \left(t\right)-\frac{1}{\tau_{e\mathrm{ff}}}\text{w}\left(t\right), \end{array}$$

where *c*_*w*_ and τ_*eff*_ denote arbitrary positive constants, aligns weights ***w*** with the result of Equation (4). Using either learning rule, the system will be excited along the principal oscillation mode of the observed movement.

### 2.2. Neural implementation of coordinate transformations

#### 2.2.1. Input transformation: plasticity

For the input transformation, we suggest a simple neural timing network, where proprioceptive input ν_φ, *i*_ from all synchronously acting muscles converges on a single postsynaptic timing neuron via synapses with weight *w*_*STDP, i*_ (cf. *timing unit* in Figure 1B). This single neuron could in nature correspond to a pool of postsynaptic neurons. Our network is based on the findings from previous experiments, which have shown that proprioceptors innervating single muscles involved in a periodic movement can adjust the timing of the motor signal that drives all muscles (Whelan et al., 1995a). Under the approximation of linear input summation, the firing rate ν_*post*_ of the postsynaptic neuron amounts to

(6) $$\begin{array}{l} \nu_{post}\left(t\right)={\text{w}}_{STDP}^{T}\left(t\right)\nu_{\varphi }\left(t\right). \end{array}$$

The efficacy of individual muscles to change the timing was found to be subject to plasticity (Whelan et al., 1995b). Assuming that the weights are subject to Hebbian plasticity combined with synaptic scaling, Oja (1982) demonstrated that the weight change of our network can be described by

(7) $$\begin{array}{lll} \frac{d}{dt}{\text{w}}_{STDP}\left(t\right) & =\gamma \left[{\text{w}}_{STDP}^{T}\left(t\right)\nu_{\varphi }\left(t\right)\right]\; & \;\\ & \times \left(\nu_{\varphi }\left(t\right)-\left[w_{STDP}^{T}\left(t\right)\nu_{\varphi }\left(t\right)\right]{\text{w}}_{STDP}\left(t\right)\right).\; & \; \end{array}$$

In case that ν_φ, *i*_ ∝ φ_*i*_, Oja's rule equals Equation (4), and our neural network would transform the input signals from the multi-dimensional joint space into the controller space, i.e., would implement Equation (1).

STDP extends the idea of Hebbian plasticity. It considers both the case of a causally related and unrelated firing of the pre- and postsynaptic neurons. In later simulations, we numerically address the question if also biologically more realistic STDP rules extract the dominant principal component of the motion.

#### 2.2.2. Output transformation: neuromodulation

For the output transformation, we model a motor feedback loop via the raphe nucleus medianus, obscurus and pallidus, which release serotonin (5-HT) into the spinal cord. The released 5-HT leads to metabotropic enhancement of motoneuron output (Heckman et al., 2008; Perrier et al., 2013). The feedback loop is based on the fact that the same nuclei receive proprioceptive information and quickly increase their firing rates with sensory input (Springfield and Moolenaar, 1983; Jacobs et al., 2002).

We assume that for each joint *i* involved in the periodic movement there is a group of serotonergic medullary neurons that receives proprioceptive input ν_φ, *i*_ via proprioceptors from a joint and project back to the motoneurons innervating this joint exclusively. Their firing rate is thus ν_*ser, i*_ = ν_φ, *i*_ (cf. *raphe neurons* in Figure 1B).

The concentration of 5-HT in the extracellular space, denoted [5-HT], increases proportionally to the firing rate of the releasing raphe neurons, ν_*ser*_ (Hentall et al., 2006; Best et al., 2010). Depletion of 5-HT can occur by reuptake into the cytosol of the cell by the *serotonin transporter* (*SERT*; denoted by *V*_*SERT*_), due to catabolism mainly by monoamine oxidase and aldehyde dehydrogenase (denoted by *V*_*cat*_), or by removal due to glia or diffusion (denoted by *V*_*rem*_) (Best et al., 2010). The rate of change thus amounts to

(8) $$\begin{array}{l} \frac{d\left[5-HT\right]}{dt}=c_{ser}\nu_{ser}-V_{SERT}-V_{cat}-V_{rem}, \end{array}$$

where *c*_ser_ is a constant.

Diffusion of 5-HT can be neglected in the spinal cord (Brumley et al., 2007). The remaining mechanisms of disappearance of 5-HT follow Michaelis-Menten kinetics,

(9) $$\begin{array}{llllll} V_{x} & =\frac{v_{max}^{x}}{\frac{k_{m}^{x}}{\left[5-HT\right]}+1}\; & & \; & \; & \; \end{array}$$

(10) $$\begin{array}{lll} & \approx \frac{v_{max}^{x}}{k_{m}^{x}}\left[5-HT\right]\;if\;\left[5-HT\right]\ll k_{m}^{x}\; & \; \end{array}$$

where $v_{max}^{x}$ denotes the maximal rate of disappearance and $k_{m}^{x}$ the respective Michaelis-constant of mechanism x (Best et al., 2010). The Michaelis constant for depletion due to reuptake by SERTs ranges between 170 and 410nM (Verleysdonk et al., 2004; Best et al., 2010), is larger than 94,000nM for catabolism (Molodtsova, 1983; Best et al., 2010), and around 400nM for glia cells (Katz and Kimelberg, 1985).

After high-frequency stimulation of raphe nuclei *in vivo*, $\left[5-HT\right]\ll k_{m}^{x}$ in the spinal cord (Hentall et al., 2006). Therefore, the approximation in Equation (10) is valid and Equation (8) reduces to

(11) $$\begin{array}{lll} \frac{d\left[5-HT\right]}{dt} & \approx c_{ser}\nu_{ser}-\left(\frac{v_{max}^{SERT}}{k_{m}^{SERT}}+\frac{v_{max}^{cat}}{k_{m}^{cat}}+\frac{v_{max}^{rem}}{k_{m}^{rem}}\right)\left[5-HT\right]\; & \; \end{array}$$

(12) $$\begin{array}{lll} & =c_{ser}\nu_{ser}-\frac{1}{\tau_{e\mathrm{ff}}}\left[5-HT\right].\; & \; \end{array}$$

Extracellular serotonin concentration in a motorpool monotonically and linearly increases the slope of the input-output function of the motoneurons (Heckman et al., 2003). Therefore, we can define multiplicative neuromodulatory weights that describe the amplification of ionotropic input as

(13) $$\begin{array}{l} w_{NM,i}=c_{NM}{\left[5-HT\right]}_{i}. \end{array}$$

Equation (12) can thus be reformulated to

(14) $$\begin{array}{l} \frac{dw_{NM,i}}{dt}={ĉ}_{ser}\nu_{\varphi ,i}-\frac{1}{{\hat{\tau }}_{e\mathrm{ff}}}w_{NM,i}. \end{array}$$

Since this is equivalent to Equation (5), our network will lead to an output transformation equivalent to Equation (3).

We suggest that both neural sub-systems finally converge on motorpools. The ionotropic input represented by ν_*post*_ is proportionally transformed into a motor signal by multiplication with a constant *m*_*f*_,

(15) $$\begin{array}{l} f_{z}\left(t\right)=m_{f}\nu_{post}\left(t\right) \end{array}$$

and the motoneurons exert a force (sliding joint) or torque (rotatory joint) on the joints *i* they innervate of

(16) $$\begin{array}{l} f_{i}\left(t\right)=w_{NM,i}\left(t\right)f_{z}\left(t\right). \end{array}$$

### 2.3. Simulations

We test the neural implementation of our algorithm using three different simulations. The first one is a simple feed-forward implementation to show that the sub-networks are able to extract the dominant mode from a large variety of sensory input. In the second closed-loop implementation, the neural network receives sensory input from and control the motor output to a linear mechanical system with known resonance behavior. This mechanical system is finally replaced by a more realistic system approximating a hopping leg. The feedback systems show that the neural network is able to induce energy-efficient movements in biomechanical systems with multiple joints and realistic ground contact situations.

#### 2.3.1. Open-loop implementation

**Figure 2:**
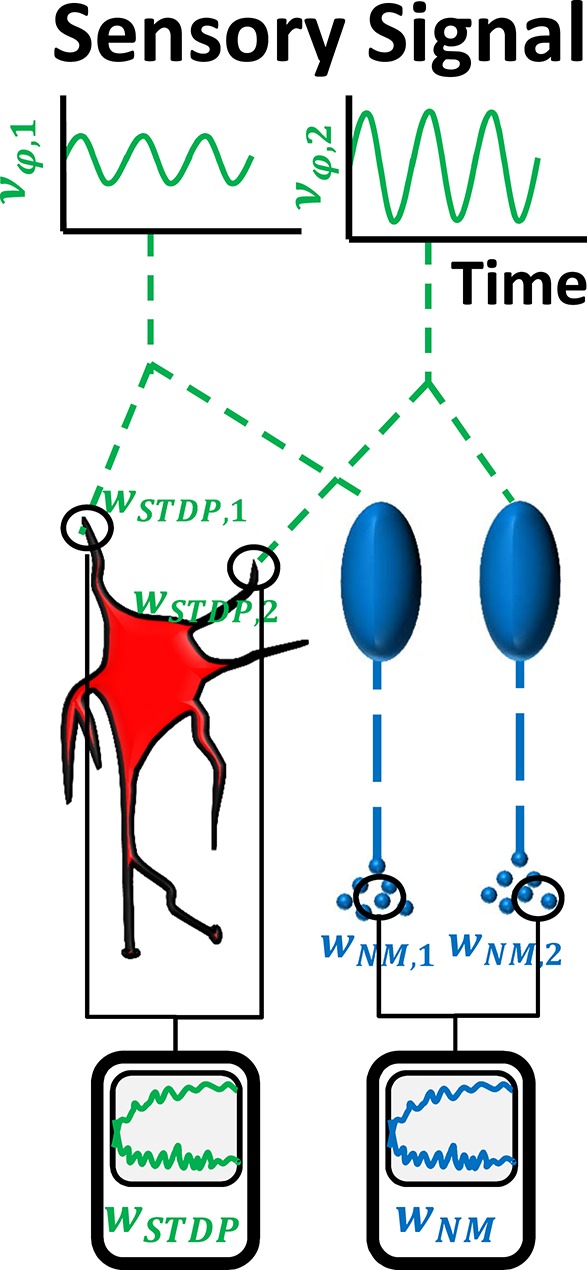
**In the first experiment, we verify the possibility to use STDP and serotonin dynamics to obtain the dominant principle component of input signals, by simulating two sensory Poisson neurons**. Shown here are their firing rates that evolve according to sinus functions ν_φ, 1_ and ν_φ, 2_ with different amplitude and underlying white noise (noise not illustrated in the picture). The neurons drive a third Poisson neuron (center left). Synaptic weights *w*_*STDP, i*_ are subject to STDP and their ratio is expected to converge toward the amplitude ratio of the input sinus functions. Additionally, the input neurons drive two raphe neurons (center right), which release serotonin into separate pools. The serotonin concentration decreases according to Michaelis-Menten dynamics. The ratio of serotonin concentrations is proportional to the ratio of the neuromodulatory weights, *w*_*NM*, 1_∕*w*_*NM*, 2_, and is also expected to converge toward the amplitude ratio of the input signals.

**Figure 3:**
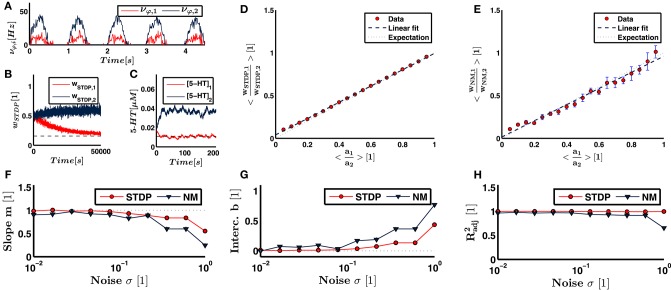
**Illustrated here are the results of the feed-forward neural network**. **(A)** The firing rate of the sensory input neurons from joint 1 and 2 for one example run with an underlying eigenmode described by the ratio *a*_1_∕*a*_2_ = 0.3. Both input and output weights are supposed to align with the dominant principal component, i.e., eigenmode. **(B)** The evolution of the synaptic input weights under the influence of STDP for the two neuron pools. The expected ratio *w*_*STDP*, 1_∕*w*_*STDP*, 2_ = 0.3 and the converged synaptic strength corresponding to joint 2, *w*_*STDP*, 2_, determine the expectation value for *w*_*STDP*, 1_. The expectation value is indicated as dashed line. **(C)** Same illustration for the serotonin concentration, which corresponds to output weighting. **(D)** The ratio of plastic input weights linearly increases with the expected ratio, i.e., *a*_1_∕*a*_2_. **(E)** demonstrates the same behavior for the neuromodulatory output weights. Theoretically predicted is a line of unit slope and zero y-intercept. **(F,G)** When the noise in the sensory input increases, the slope and intercept of the linear fit increasingly deviate from the expectation. **(H)** Even for large noise levels, we find an adjusted *R*^2^ value close to 1. This indicates that our linear fits, and hence the obtained values for the slope and y-intercept, remain reasonable.

The open-loop feed-forward implementation is comprised of two sensory neurons which are connected to a postysnaptic timing neuron and to the parallel serotonergic feedback system (cf. Figure 2). Each sensory neuron represents the pool of proprioceptive neurons responsible for one joint *i*. The individual neurons fire according to Poisson statistics with mean firing rates ν_φ, *i*_ which oscillate in phase with different amplitudes *a*_*i*_ (cf. Figure 3A). To test if the system is robust against disturbances, we add Gaussian white noise ***n***(σ) with standard deviation σ = 0.1 to the sensory input. An additional sinusoidal contribution ***b*** of Euclidean vector norm smaller than ***a*** simulates a secondary eigenmode of the biomechanical system. The firing rates thus amount to

(17) $$\begin{array}{l} \left(\begin{array}{c} \nu_{\varphi ,1}\left(t\right)\\ \nu_{\varphi ,2}\left(t\right) \end{array}\right)=40Hz\left[\left(\begin{array}{c} a_{1}\\ a_{2} \end{array}\right)\sin\left(2\pi t\right)+\text{b}\sin\left(8\pi t\right)+\text{n}\left(\sigma \right)\right]. \end{array}$$

In the timing sub-network, the sensory neurons are directly connected to a third Poisson-neuron that represents the timing unit. This postsynaptic neuron fires with a rate of

(18) $$\begin{array}{l} \nu_{post}\left(t\right)={\text{w}}_{STDP}^{T}\left(t\right)\nu_{\varphi }\left(t\right). \end{array}$$

The synaptic weights are subject to an STDP rule that is based on previous experiments (Pfister and Gerstner, 2006) which considered the effect of spike triplets (e.g., two pre- and one postsynaptic spike). We stabilize the weights using synaptic scaling as homeostatic mechanism (cf. Section 4.2).

Each sensory neuron is connected to a corresponding raphe neuron. Spikes of each raphe neuron increase the serotonin concentration in a respective pool. The concentrations [5-HT]_*i*_ in the two pools *i* decrease according to Michaelis-Menten kinetics. Our derivation, which shows that serotonergic dynamics can extract the dominant principle component, assumes that $\left[5-HT\right]\ll k_{m}^{x}$ (cf. Equation 10). Therefore, simulations implementing a small value for the Michaelis constant represent the strongest validation of our derivation. We choose the smallest Michaelis constant suggested by the literature mentioned in Section 2.2.2: *k*_*m*_ = 170*nM*.

The vector of input weights ***w***_*STDP*_ and output weights ***w***_*NM*_ should converge toward ${\left(a_{1},a_{2}\right)}^{T}$. We simulate the neural network with 19 different ratios $\frac{a_{1}}{a_{2}}$ ranging between 0.05 and 0.95 and set ∥a∥ = 1. Both ***w***_NM_ = *c*_NM_ ${\left({\left[5-\text{HT}\right]}_{1},{\left[5-\text{HT}\right]}_{2}\right)}^{T}$ and ***w***_*STDP*_ are supposed to align with the eigenmode. This implies $\frac{w_{\mathrm{NM},1}}{w_{\mathrm{NM},2}}=\frac{w_{\mathrm{STDP},1}}{w_{\mathrm{STDP},2}}=\frac{a_{1}}{a_{2}}$. We hence fit the converged ratio $\frac{w_{\mathrm{NM},1}}{w_{\mathrm{NM},2}}$ and $\frac{w_{\mathrm{STDP},1}}{w_{\mathrm{STDP},2}}$ vs. $\frac{a_{1}}{a_{2}}$.

Figures 3B–E illustrate the convergence of weights. The ratio of input weights are best fit by a line described by

(19) $$\begin{array}{l} \frac{w_{\mathrm{STDP},1}}{w_{\mathrm{STDP},2}}=\;m\frac{a_{1}}{a_{2}}+b, \end{array}$$

(20) $$\begin{array}{l} m=\;0.952\pm 0.005 \end{array}$$

(21) $$\begin{array}{l} b=\;0.040\pm 0.003 \end{array}$$

(22) $$\begin{array}{l} R{}_{\mathrm{adj}}^{2}=\;0.999. \end{array}$$

$R_{\mathrm{adj}}^{2}$ denotes the adjusted *R*^2^-value. We obtain similar findings for the neuromodulatory weights,

(23) $$\begin{array}{lll} \frac{w_{\mathrm{NM},1}}{w_{\mathrm{NM},2}} & =\; & \;m\frac{a_{1}}{a_{2}}+b, \end{array}$$

(24) $$\begin{array}{l} m=\;0.945\pm 0.033 \end{array}$$

(25) $$\begin{array}{l} b=\;0.015\pm 0.019 \end{array}$$

(26) $$\begin{array}{l} R{}_{\mathrm{adj}}^{2}=\;0.979. \end{array}$$

To test the influence of the initial conditions, we run nine additional trials with random initial synaptic weights and serotonin concentrations. Averaging the parameters over all ten trials yields

(27) $$\begin{array}{ll} & m=0.979\pm 0.010 \end{array}$$

(28) $$\begin{array}{l} b=0.016\pm 0.002 \end{array}$$

for synaptic weights while we obtain for neuromodulatory weights

(29) $$\begin{array}{ll} & m=0.957\pm 0.031 \end{array}$$

(30) $$\begin{array}{l} b=0.005\pm 0.023. \end{array}$$

Our theoretical considerations predict a slope of *m* = 1 and an intercept of *b* = 0. The slope representing both synaptic and neuromodulatory weights and the intercept of synaptic weights deviate from the expectation values by several standard deviations. The deviations are thus small, but significant. Under the influence of white noise, Oja's rule, Equation (4), converges toward the dominant principal component of input data (Oja, 1982), which is equivalent to the dominant eigenmode in a linear system (Feeny and Kappagantu, 1998). Hence, the deviations of the slope and intercept from their expectation values derive on the one hand from the minor eigenmode and the Poisson noise underlying the neural firing statistics, and on the other hand from the deviations between calculations performed by the implementation of STDP and Michaelis-Menten kinetics from Oja's rule.

To show robustness against sensory noise, we vary the standard deviation of the Gaussian white noise in the sensory input. We sweep through a range of σ between 0.01 and 1.0. Figures 3F–H illustrate the slope, y-intercept and adjusted *R*^2^ value for each noise level. We see that the linear approximation remains valid for high noise levels, as represented by a $R_{\mathrm{adj}}^{2}$ close to unity at σ = 1. The slope and y-intercept increasingly deviate from the expectation for higher noise levels. However, the slope and intercept representing synaptic and neuromodulatory weights deviate by less than 10% from the expected value for noise levels σ < 0.2 and σ < 0.13, respectively. Since the dominant eigenmode ***a*** is normalized, a value of σ = 0.1 implies that the firing frequency of any sensory or the postsynaptic neuron is influenced by noise by more than 10% on average. Thus, within a given time step, the probability that either an occurring neural spike is due to noise or that a neural spike is inhibited because of noise is higher than 10%. These results suggest a strong robustness of neural calculations performed by our network against noise.

#### 2.3.2. Closed-loop implementation

**Figure 4:**
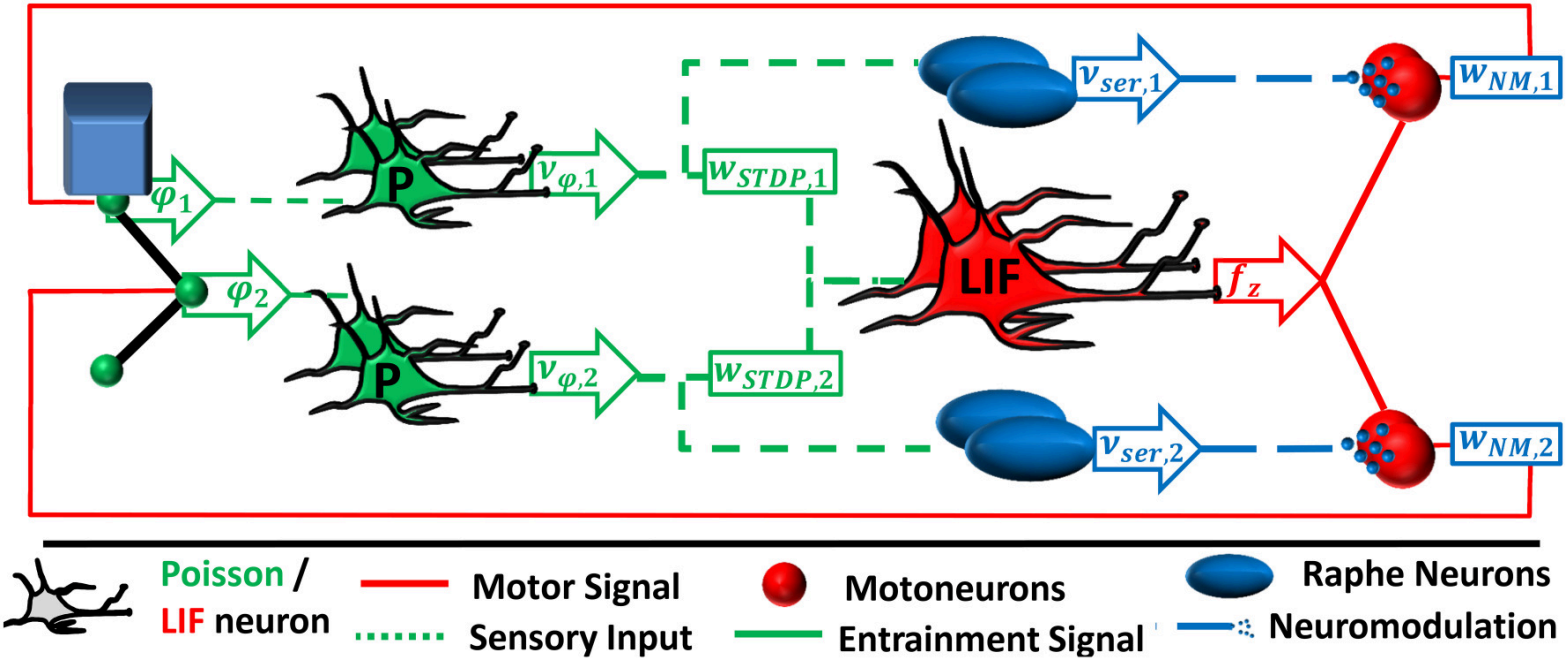
**The neural feedback network is an extended version of the feed-forward network as described in Figure 2**. In contrast, the spinal interneurons are implemented as leaky integrate-and-fire (LIF) neurons. Their pool averaged firing rate is proportionally transformed into a motor signal *f*_*z*_. The motor signal is amplified by a factor *w*_*NM, i*_ due to serotonergic neuromodulation and is projected back to the muscles.

In order to test the ability of our neural controller to drive a mechanical system with multiple degrees of freedom, we simulate the complete neural network in a closed-loop feedback system (cf. Figure 4).

We implement the two neural sub-systems in parallel, each receiving proprioceptive input. The deflection of each joint is signaled by a pool of Poisson neurons firing with an average rate proportional to the deflection. The sensory neurons are connected to a pool of leaky integrate-and-fire (LIF) neurons. Synaptic weights are subject to the same STDP rule as described above. Since the instantaneous pool-averaged firing rate of the LIF neurons, ${\bar{\nu }}_{\mathrm{post}}$, serves as ionotropic input to the motoneurons, the neuron pool functionally represents the timing and the driving unit (cf. Figure 1B).

The sensory neurons of each individual joint are additionally connected to a respective pool of serotonergic raphe nuclei. The raphe nuclei are also composed of Poisson neurons. Every spike of a raphe neuron releases 5-HT into the corresponding motorpool. Within an individual motorpool, the release is spatially uniform. Depletion takes place according to Michaelis-Menten kinetics. Once again, we choose the smallest suggested Michaelis constant. The resulting [5-HT] is here given in units of mol/l= M. The motoneuron firing rate is amplified proportionally to [5-HT] (Heckman et al., 2003).

**Figure 5:**
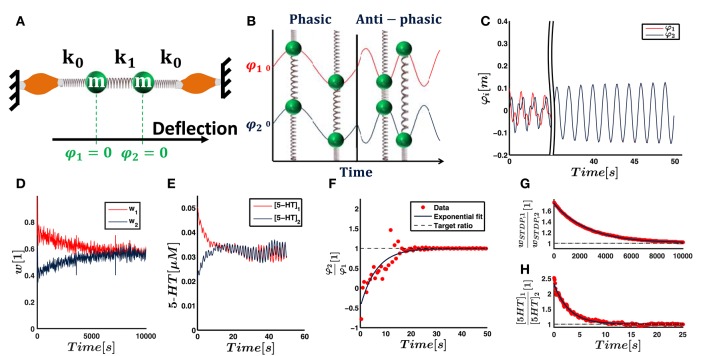
**The neural network as described in Figure 4 is initially supposed to control a simple mechanical system**. **(A)** Two masses *m* are connected by springs of stiffness *k*_0_ and *k*_1_ and driven by muscles that can stretch the springs. Zero positions are given by the equilibrium positions when no force is applied. The deviations of the masses from their zero positions are used as joint deflections φ_*i*_. The neural network as described in Figure 4 controls the muscles' forces. **(B)** The system has analytically known resonance modes of (1, 1)^*T*^ and (−1, 1)^*T*^, i.e., the masses either oscillate in phase or anti-phasic. The task of our controller is to excite the system along any of the two eigenmodes with corresponding respective eigenfrequency. The other eigenmode decays due to friction. The final movement is thus resonant. **(C)** The deflection trajectories of both joints align and show phasic resonant movement after few seconds. **(D)** shows that the input weights, which are subject to STDP, converge toward the same value within hours. They therefore also align with the phasic resonance mode (1, 1)^*T*^ of the mechanical system. **(E)** shows that the 5-HT concentration within both motoneuron pools, and hence the output weights, converge toward the same value within seconds. **(F)** The alignment of the trajectories is illustrated by the deflection ratio φ_2_∕φ_1_ at peak positions of mass *m*_1_. Shown here is the time evolution of this ratio and an exponential fit. **(G,H)** The ratio of the synaptic and neuromodulatory weights converge to unity with different time scales, as illustrated by respective exponential fits. (All results illustrate the simulation with non-random initial weights).

**Figure 6:**
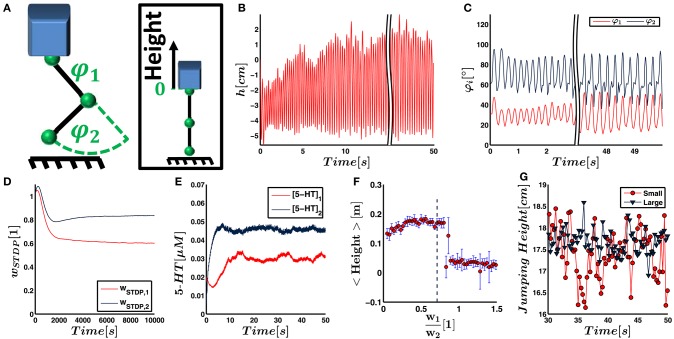
**(A)** In the final simulation, the neural network as described in Figure 4 is designed to control a more biologically realistic trunk model that has a jumping leg with two joints influenced by gravity. The neural network gets sensory input about joint deflections and controls the torque within these joints. The jump height is taken as indicator for energy efficiency. It is measured as height of the trunk relative to its position when both joints are fully extended. **(B)** The jump height of the leg increases as a result of the weight adjustment by the neural network. **(C)** The joint deflections are linearly converted to firing rates of the sensory neurons and therefore also represent the sensory input to the neural network. **(D)** The synaptic weights from proprioceptors innervating muscles of joint 1 and 2 to the pool of postsynaptic LIF neuron converge on a time scale of hours. **(E)** Neuromodulatory weights are proportional to the serotonin concentration. They converge faster than the synaptic weights, and the serotonergic concentration within the two motoneuron pools starts to fluctuate around its final value after seconds. **(F)** To find conditions for energy efficiency of the controller numerically, we fix the Euclidean norm of the weight vectors describing the synaptic input and neuromodulatory output weights. We vary the ratio *w*_1_∕*w*_2_ both for input and output weights, run a separate simulation for each ratio and record the respective jump height. As illustrated, the jump height has a maximum plateau for ratios between 0.4 and 0.75. This ratio is in agreement with the weight ratios obtained by STDP and serotonin dynamics. **(G)** Even after convergence, the jump height, illustrated as maximum height above the ground here, shows fluctuations with time. We suggest that this is due to noise and the small number of leaky integrate-and-fire neurons that is intrinsic to the controller network. Accordingly, increasing the network size from small to large significantly decreases the fluctuations. (All results illustrate the simulation with non-random initial weights).

We consider two mechanical systems; one is simple and analytically solvable (cf. Figure 5A), the other more complex and biologically realistic (cf. Figure 6A).

The first system consists of two masses, each representing one joint, that are serially coupled by a spring. Each mass is connected to muscles by further springs of equal stiffness. The system is driven by forces *f*_*i*_ of the muscles, which stretch and squeeze the springs. As illustrated in Figure 5B, the system is analytically known to follow the eigenmodes (1, 1)^*T*^ (phasic oscillation) and (1, −1)^*T*^ (anti-phasic oscillation).

Figures 5D,E shows that the weights of both joints converge toward the same values. This corresponds to phasic resonance movements of the two joints. Fitting exponential functions to the ratio of $\frac{w_{\mathrm{STDP},1}}{w_{\mathrm{STDP},2}}\left(t\right)$ and $\frac{w_{\mathrm{NM},1}}{w_{\mathrm{NM},2}}\left(t\right)$ shows that the input and output weights converge toward unity with exponential time constants of τ_*STDP*_ = 2.65 × 10^3^s and τ_*NM*_ = 2.93s (cf. Figures 5G,H). These time constants differ by three orders of magnitude and we refer to Section 3.1.1 for a discussion of the different time scales. As shown in Figure 5C, the resulting motion trajectory of the mechanical system starts as a superposition of its eigenmodes, showing no obvious relationship in phase or amplitude between the two masses. The trajectories of the two masses converge to synchronous resonant movements over time. Fitting an exponential function to the ratio of joint deflections $\frac{\varphi_{2}}{\varphi_{1}}\left(t\right)$ at peaks of the first joint deflection, we find that this synchronization takes place with a time constant τ_*traj*_ = 6.24s (cf. Figure 5F).

To test the dependence of our results on the initial conditions, we randomly vary the initial synaptic weights and serotonin concentrations in nine additional trials. As an average over all ten trials, we find

(31) $$\begin{array}{l} \frac{w_{\mathrm{STDP},1}}{w_{\mathrm{STDP},2}}=1.005\pm 0.011 \end{array}$$

(32) $$\begin{array}{l} \frac{w_{\mathrm{NM},1}}{w_{\mathrm{NM},2}}=1.004\pm 0.032. \end{array}$$

These values agree with our expectation of unit weight ratios.

The second mechanical system represents a vertically jumping leg consisting of a trunk, a thigh, and a shank, which are connected by rotatory hip and knee joints. When the leg touches down, the joints are deflected, which leads to a stretching of the elastic elements. This stretching triggers the firing of the sensory neurons and activates the reflex arc. The respective torque *f*_*i*_ is exerted on the joint *i* according to Equation (16) and the leg pushes off the ground.

Figures 6D,E demonstrate for one run that the input and output weights converge on a time scale of seconds and hours, respectively. A video illustrating this simulation can be found in Video 1. Again, we perform nine additional runs to test for stability against changes of the initial conditions. As an average over all 10 simulations, we obtain

(33) $$\begin{array}{llll} \frac{w_{\mathrm{STDP},1}}{w_{\mathrm{STDP},2}}=0.724\pm 0.021 & \; & & \end{array}$$

(34) $$\begin{array}{l} \frac{w_{\mathrm{NM},1}}{w_{\mathrm{NM},2}}=\;0.703\pm 0.062. \end{array}$$

The jump height increases with time and reaches its maximum within seconds (cf. Figures 6B,C). The increasing jump height indicates that the movement is tuned to yield higher energy efficiency. To validate that the network increases the energy efficiency of the movement, we run the simulation with different fixed input and output weights. In each trial, we set the norm of the weight vectors equal to the converged norm as obtained in the trial illustrated in Figures 6B–E. We only vary the ratio of the weights, i.e., the orientation of the weight vector. Figure 6F illustrates that the final jump height has a maximum plateau for weight ratios $\frac{w_{1}}{w_{2}}$ between 0.4 and 0.75.

We analyze the alignment of our weights with the dominant principal component of the movement. For this analysis, we extract the dominant principal component *a* of the joint trajectory φ(*t*) for each of the ten runs. The average ratio amounts to $\frac{a_{1}}{a_{2}}=0.767\pm 0.027$. In comparison to the initial synaptic weights, the converged synaptic weights are closer to the ratio of the principal dominant component in 9 out of 10 trials. Assuming that this alignment happens by chance, we would expect to see alignment on average in 50% of the runs. This hypothesis can be rejected by a probability *p* < 0.05. For the serotonin concentration in the two motorpools, we find alignment in 10 out of 10 runs, indicating *p* < 0.001.

As shown in Figure 6B, the peak jump height does not converge but shows fluctuations. Considering the average in the window of 30–50 s of simulated time, the jump height shows a standard deviation of 5.8mm. A possible reason for the fluctuations is the intrinsic (Poisson) noise in the system and a relatively small number of only six LIF controller neurons which is chosen due to computational restrictions. To test the influence of noise and the network size, we increase the number of sensory input neurons by a factor of 3 and the number of LIF neurons by a factor of 2. For reasons of comparison, we keep the strength of the input to the LIF neurons as well as the motor signal approximately equal. As a result, we decrease the initial synaptic weights and the neuromodulatory amplification factor *c*_*NM*_ by the same respective factors. The standard deviation of the jump height decreases to 2.7mm (cf. Figure 6G). The 76 measurement points of jump height recorded for either network do not show any outliers, are not significantly correlated with time, and do not significantly deviate from a normal distribution. They therefore fulfill the requirements to test for different standard deviations using an *F*-test. The test shows that the standard deviation in the jump height controlled by the large and small network deviate by *p* < 0.001. Therefore, a larger network size and reduced sensory noise decreases fluctuations in the joint trajectories.

## 3. Discussion

In this study, we consider how the CNS may coordinate fast periodic movements involving several joints. We propose a simple algorithm for this task and confirm that a neural implementation, which is consistent with previous experiments, may explain the correct inter-joint coordination between joints that act with high phase synchronicity as observed for explosive movements. The controller excites the mechanical system along the dominant local eigenmode by a coordinate transformation of proprioceptive inputs from the joint space into a one-dimensional controller space and an inverse transformation of driving controller output. The eigenmode is recursively extracted from the proprioceptive input describing the movement.

We demonstrate that this weighting can be performed by a small network of sensory afferents that converge onto a common pool of spinal interneurons via plastic synapses. Similarly, we reason that a motor feedback loop from proprioceptors via medullary serotonergic neurons may approximate the appropriate output weighting.

### 3.1. Advantages of the controller design

#### 3.1.1. Stability and energy efficiency

Our controller design generates stable and energy-efficient periodic movement. In previous research, we have demonstrated that the basic controller design can induce stable movements in robotic platforms (Lakatos et al., 2013b; Lakatos and Albu-Schäffer, 2014a,b). In our simulations, the neural implementation can also induce stable movements in two mechanical systems of different complexity. The induced movement is stable over hours for both the linear and the non-linear mechanical system, as tested for a large range of initial conditions. Our results emphasize that the two neural sub-networks reliably extract the dominant principle component of sensory input signals even in the presence of different disturbances. In particular, we tested stability against noise and perturbations resulting from excitation of a second eigenmode. The converged weights did not fully align with the dominant principle components since their calculations deviate from the mathematical controller design due to different biological features. These firstly include signal transduction durations as included by delays of sensory signals in the network driving the mechanical leg (cf. Section 4.4.2). Second, the mathematical descriptions of STDP and neuromdoulation are based on experimental measurements and deviate strongly from Oja's rule, which underlies the robotic controller design. This deviation is increased by the fact that the spiking of sensory and the postsynaptic neurons in the feed-forward simulation bear Poisson noise. Third, the input-output function of spiking LIF neurons deviate from the basic bang-bang controller (defined by Equation 2) as described in the discussion in Section 4.4.2. Another reason for the deviation in the feed-forward simulation comes from the fact that we added a secondary minor eigenmode to the sensory firing rates to test for stability against disturbances. Despite these constraints, the feed-forward simulations show that a strong alignment of weights with the dominant principal component does take place. Extending the sample size of simulations with different initial conditions, levels of noise and disturbances would quantify more precisely the level of alignment. But a value quantifying the alignment of weights in a feed-forward simulation under a limited variety of disturbances results only indirectly in a statement about the ability of the neural network to control biomechanical systems, which may show an arbitrary variation of disturbances, in a feedback loop. Thus, a more precise quantification of the alignment, i.e., more trials, would only yield little advantage. In the feedback system where the neural network controls the simple mechanical system, the weights seem to align even more reliably with the theoretical expectation. Here, an increased number of neurons decreases the relative influence of Poisson noise in the sensory input. Additionally, the final movement can be fully described by a single eigenmode of the system. Therefore, the sensory signals are not disturbed by a secondary eigenmode in the end of the simulation. With the biological constraints still in place, these factors lead to a better agreement between expectation and theory in the simple feedback simulation.

Tuning of the movements to increase energy efficiency by our controller design is strongly linked to linearization of the mechanical system. The dominant principle component of a movement is equivalent to the eigenmode of a linear mechanical system associated with the largest eigenvalue of the covariance matrix (Feeny and Kappagantu, 1998), i.e., the eigenmode that best describes the observed movement trajectories. An eigenmode that is e.g., only lightly damped and close to the initial weights is likely to dominate the overall movement and the controller will favor to excite this eigenmode over others. Our controller hence aligns the transformation weights with one of the eigenmodes, (1, 1)^*T*^ in our simulations, and thereby obtains resonance tuning for systems such as our simple mechanical model.

For non-linear systems such as our leg, this is not necessarily the case. However, our controller design assumes that energy is only inserted into the system by the bang-bang controller during a relatively short period of the movement cycle. It is therefore reasonable in practice to compute the control action based on the linearization of the non-linear system at the current state. Our simulation of the mechanical leg emphasizes this point. The synaptic input and neuromodulatory output weights start from random initial values in the range of 0.67–1.5 for relative weights $\frac{w_{\mathrm{STDP},1}}{w_{\mathrm{STDP},2}}$ and 0.1–10 for $\frac{w_{\mathrm{NM},1}}{w_{\mathrm{NM},2}}$, respectively. The weight ratio reliably converges toward a value of about 0.7. Figure 6F illustrates the jump height as a function of the ratio $\frac{w_{\mathrm{STDP},1}}{w_{\mathrm{STDP},2}}=\frac{w_{\mathrm{NM},1}}{w_{\mathrm{NM},2}}$. It shows that the value of 0.7 is clearly in the range of ratios that maximize the jump height. In Section 4.7.3, we explain that this finding implies tuning to energy efficiency. These results suggest our controller design and our neural models in particular as a candidate to explain how the CNS may excite stable and energy-efficient fast periodic movements. We are currently conducting further testing to analyze in detail the conditions that allow our controller to increase energy efficiency.

Our neural sub-networks show that the control algorithm may be implemented by two spatially separated units. One unit consists of the ionotropic sensory neurons and spinal interneurons and acts at the sensory input level of the spinal cord. A second unit performs individual amplification of the motor signal for each joint. We consider the role of each unit individually.

The output amplification, i.e., the second unit, is necessary for energy-efficient control. If the two masses of our linear mechanical system were excited with forces of different amplitude, the mass trajectories would never converge to resonant movement. This agrees with the fact that the output weights and joint trajectories in our feedback simulations converge on a similar time scale.

The weights in the input network in contrast converge on a slower time scale of hours. They can therefore not react to quick changes of the environment, but to slow biomechanical changes. We intentionally set this slow time scale in agreement with experiments on STDP *in vivo* (e.g., Nishimura et al., 2013; cf. Section 4.2). The discrepancy between this time scale and the fast convergence of joint trajectories as found in our simulations can be explained by the mentioned high synchronicity of joint motions in the considered fast periodic movements. The input weighting determines how strongly sensory input from each joint participates in the entrainment of motor output. If all joints would move in phase, the motor output could be entrained to an arbitrary linear combination of the sensory input. Therefore, the input weighting is not strictly necessary for energy-optimal tuning. In contrast to an approach where motor output is entrained to the signal of only a single nerve, our input network would have three features better suited for animals. First, the motion of joints in biomechanical systems will not be exactly in phase. In this case, our controller gives higher priority to the timing of muscles that are more important. Second, our network gives higher efficacy to nerve fibers that fire more strongly. Under the influence of additive noise, higher activity is connected to a better signal to noise ratio. Thus, entrainment is mainly affected by nerves that show the highest signal to noise ratio. Third, considering all sensory inputs reduces the risk to failure, e.g., when individual nerve fibers are damaged.

To summarize, output weighting by the CNS is required for energy-optimized movement. The fast time scale of neuromodulation may thus allow animals to quickly adjust their movements to changes in the environment. In contrast, the CNS must not necessarily implement the input stage of our controller. However, since the input transformation is advantageous, it is plausible that the neural timing network that we propose may adjust weighting on a longer time scale to compensate for slow mechanical changes.

#### 3.1.2. Dimensionality reduction

The design of our proposed neural network implies characteristic features of the functional driving unit of considered movements (cf. Figure 1B). In our simulations, the pool of timing neurons functionally represents also the driving unit and form a reflex arc.

The driving unit is effectively one dimensional. It receives input from all joints and sends the same motor signal to all motorpools. This single signal is weighted by the neuromodulatory weights to project the one dimensional controller signal back into joint space. At the stage of the timing unit, the input and output weights have thus transformed the control of the mechanical systems to a one-dimensional problem.

This is obvious for the control of the simple mechanical system, which only comprises a single reflex interneuron to create the motor signal to all joints. However, the neural controller of the mechanical leg has six timing neurons. Nonetheless, they act as a single functional unit. The reason is that each timing neuron receives input from a large pool of sensory neurons from each joint. Each sensory neuron has the same probability to connect to any of the six timing neurons, and the pools of sensory neurons projecting to the individual timing neurons largely overlap. The motor signal is furthermore averaged over all six timing neurons, and the same signal is transferred to both motorpools, where it is amplified by neuromodulation. Therefore, the control is still transformed to a functionally one-dimensional problem despite the existence of the six timing neurons. There are three reasons why we decided for a neuron pool instead of a single timing neuron: First, it makes the model more realistic. Second, it reduces the influence that Poisson noise in the sensory neurons has on synaptic weights. Third, it smooths the output signal of the pool of LIF neurons.

#### 3.1.3. Interplay of reflexes and CPGs

Although not considered in our simulations, the timing and driving unit may be spatially separated. The driving unit must produce rhythmic output that is phase-coupled to the output of the timing unit. For example, Xiong et al. (2015) and Buchli and Ijspeert (2008) proposed CPG models that fulfill a task similar to our driving unit. It is alternatively possible that the one-dimensional task is achieved by a parallel combination of a CPG and reflex arcs that are both entrained by the timing signal and converge onto or prior to the motoneurons. The CNS may tune the relative contribution of our proposed reflex arc and a parallel CPG according to a secondary task. For example, in the beginning of a periodic movement, the reflex arc may be more active in order to react to unforeseen perturbations. When the periodic movement remains unperturbed for a longer period of time, the contribution of the CPG may increase. The serotonergic feedback network acts on the motorneurons and could thus adjust the relative strength of the motor signal without affecting the driving unit itself.

### 3.2. Biological considerations

#### 3.2.1. From joint to muscular level

Our neural controller design acts on a joint level due to its origin in robotics control theory. In animals, proprioceptive input originates from individual muscles, and the motor signal also exerts force on a muscular level. We assume that in the CNS the neural implementation of our controller would adjust weights of individual muscles and not joints. If two muscles of the control loop need to equally assist in a given movement to tune it to yield high energy efficiency, e.g., because the joints that they actuate are equally important for a given movement, they would be assigned similar weights. Antagonistic muscles would be assigned weights of opposite sign. In our simulations, weights are adjusted according to sensory signals representing joint deflections. Corresponding signals on a muscular level, which would represent muscle length, may originate in type II nerve fibers.

#### 3.2.2. Model validity

Although the CNS may use different mechanisms for the implementation of our proposed control algorithm, our neural models are based on substantiated experimental observations.

Our sub-network for input weighting is based on the finding that proprioceptive nerve fibers from leg muscles converge in the spinal cord (Jankowska, 1992), and that the stimulation of individual fibers in decerebrate cats can change the timing of all muscles involved in a movement (Whelan et al., 1995a; Hiebert et al., 1996; Rossignol et al., 2006). Circuits underlying this behavior seem to reside fully in the spinal cord (Conway et al., 1987; Hiebert et al., 1996). The efficacy of fibers from individual muscles to cause entrainment undergoes use-dependent plastic changes. The efficacy of the fibers positively corresponds to the level of their participation in the entrainment (Whelan et al., 1995b). This agrees with Oja's rule, which underlies our controller design. We suggest to link these findings with STDP, which has been reported in the spinal cord of animals at various ages (Kim et al., 2003; Schouenborg, 2004; Nishimura et al., 2013).

Our hypothesis about the serotonergic sub-network performing output weighting is comprised of a motor feedback loop via the raphe obscurus, pallidus and potentially medianus. We propose this feedback loop in Section 1 based on a large range of experimental evidence (Veasey et al., 1995; Bennett et al., 1998; Hultborn, 1999; Jacobs et al., 2002; Heckman et al., 2008; p. 46f). Using theoretical considerations, we demonstrate that serotonin dynamics can be approximated by Equation (5) and show that this equation is equivalent to Oja's rule under physiologically reasonable conditions. Therefore, it is plausible that 5-HT dynamics extract the dominant principle component of sensory input onto serotonergic neurons. Our simulations emphasize that 5-HT produces enhancement of the motoneuron output along the dominant principal component of the movement. In agreement with our simulation results, the time scale of motoneuron excitability following raphe stimulation is of the order of several seconds (Perrier and Delgado-Lezama, 2005).

The precision of sensory input to the raphe nuclei and serotonergic output onto motoneurons is a matter of current debates (Hyngstrom et al., 2007; Heckman et al., 2008; Johnson and Heckman, 2014). Our proposed serotonergic network would require the topography of the feedback arc to be at least joint-specific. This is reasonable for somatosensory input to the raphe nuclei obscurus, pallidus and medianus, since it has a delay of about 20 ms (Springfield and Moolenaar, 1983). Such a short delay favors a neural pathway with few synapses, maybe bypassing the cerebellum as has previously been described for somatosensory input to other brain stem nuclei (Landgren and Silfvenius, 1971; Johansson and Silfvenius, 1977). There are different indications for topography in spinal projections of the raphe nucleus (Skagerberg and Bjorklund, 1985; Bacon et al., 1990; Cope, 2001; Brumley et al., 2007; Perrier et al., 2013; p. 53). Sufficient topographic precision is plausible; whereas other raphe nuclei project to areas throughout the whole brain and release serotonin in a paracrine manner, projections from the considered raphe nuclei project primarily to the spinal cord (Jacobs et al., 2002; Nieuwenhuys et al., 2007, p. 896) and form well-defined synaptic connections on motoneurons (Perrier et al., 2013).

Furthermore, experiments on the level of neural networks agree with our hypothesis for the functional consequence of serotonergic modulation of motoneuron excitability. Cats show walking patterns which lack refinement after their spinal cord is transected, but not if only influence from the cerebral cortex is cut off (MacKay-Lyons, 2002). The lack of cortical influence in humans was shown for reflex modulation during explosive movements, i.e., those that benefit from the elastic dynamics, in contrast to precision tasks (Shemmell et al., 2009). Our proposed algorithm observes arbitrary movements and tunes inter-muscular coordination accordingly. In Section 3.2.1, we suggest that the PCA algorithm of our basic controller principle would assign weights of opposite sign to antagonistic muscles. The antagonistic muscles would be excited with a phase shift of 180°. In biological terms, this means that the motor signal of these two muscles (measured by EMG) would become anti-correlated over time. Our hypothesis suggests that blockade of 5-HT_2_ receptors, which are assumed to be responsible for enhanced motoneuron excitability upon 5-HT application (Sławińska et al., 2014), will disrupt this tuning. Pearlstein et al. (2005) observed exactly this behavior in rats when measuring the ventral root activity of antagonistic muscles acting on the same limb. Upon addition of a 5-HT_2_ antagonist, the cyclic movement continued while the correlation coefficient of motor signals in the ventral roots of antagonistic muscles changed highly significantly from a negative to a positive value (Pearlstein et al., 2005, Figure 5).

#### 3.2.3. Movement initiation

Since our controller design represents a reflex arc, it can only shape an ongoing but not initiate a new periodic movement. In our simulations, the mechanical systems move because they start from an imbalanced initial position. In nature, the CNS must initiate the movement with an intrinsically produced motor signal that is sent to all motor neurons. In our simulations, the relative strength of the first motor signals produced by the reflex arc are randomly chosen. Therefore, a motor signal that initiates the periodic movement does not need to be specifically tuned, either. It is sufficient to send an appropriately strong motor signal to all joints involved in the movement. This motor signal may originate in cortical areas. A CPG that may functionally replace or support the reflex interneurons at intermediate spinal levels, as proposed in Section 3.1.3, is an alternative explanation for movement initiation.

### 3.3. Implications for research

Our proposed neural sub-networks link different experimental findings into a coherent framework. Their validation would require to show that the repeated passive movement of a single joint increases the motoneuron excitability of corresponding muscles exclusively. The change in excitability must be due to 5-HT.

Our simulation results show that the presented concept of an adaptive coordinate transformation between joint and controller space is a promising hypothesis for neural calculations. While our sub-networks are plausible, we must emphasize that alternative neural implementations of our algorithm may exist and we encourage other ideas for neural interpretations. The controller design provides experimenters with guidelines for a neural circuit to search for. In the following, we provide a check list of characteristics that circuits must provide in order to tune periodic movements according to our algorithm. The circuits must

- adjust motor output for the whole limb during fast periodic movements based on proprioceptive signals.
- scale the output of motor signals to individual motoneuron pools. The relative strength of muscles must be amplified when the joint they act on shows larger deflections during the movement.
- average sensory input representing joint deflections on a time scale of seconds. This time scale must be sufficiently fast to react to environmental changes, but significantly longer than the cycle duration of the movement to prevent substantial variations during the cycle.
- include a function that keeps the strength amplification bounded. In addition, the mechanism itself must not alter relative amplifications between the muscles.
- not alter the frequency of the motor signal.

As discussed in Section 3.1.1, adjustment of the input transformation is not strictly necessary, but advantageous from viewpoint of convergence to energy-efficient fast periodic movements. A circuit that implements the input transformation must

- receive sensory information from several joints that converge onto a single functional unit. This unit must influence muscles in the whole limb.
- entrain the output frequency of this driving unit to proprioceptive signals. Hereby, the relative entrainment efficacy of a signal must be amplified when the corresponding joint shows larger deflections during the movement.
- change the relative efficacy of a signal based on the sensory information about the joint deflections as averaged on a time scale of at least seconds. This lower boundary on the time scale is in contrast e.g., to the typically short time scale of influences by a single ionotropic input. It prevents substantial variations of the weights during the movement cycle. As discussed in Section 3.1.1, there is no strict upper boundary for this time scale.
- include a mechanism that keeps the efficacy bounded, i.e., prevent runaway behavior. The mechanism must not alter the relative efficacy between the muscles.

In contrast to these requirements on the sensory input and motor output stage of the spinal cord, our algorithm and the proposed neural implementations place minimal restriction on circuits generating the ionotropic motor signal of a whole limb. Our controller design provides a driving circuit with an entrainment signal that is continuously optimized for local eigenmodes of the controlled mechanical system under changing environmental and biomechanical conditions. Similarly, it achieves correct inter-joint coordination of the motor output. Since the eigenmode is determined by the mechanical system, our controller effectively adjusts movements to biomechanical and environmental properties. As discussed in Section 3.1.3, our proposed controller and network may thus effectively simplify the dynamical interplay of CPGs and reflexes in explosive periodic movements to a one-dimensional problem.

Our results emphasize the benefits of control strategies for bio-mimicking robotic systems derived by engineering considerations, which can be well verified experimentally. We suggest that neuroscientific research can use these strategies as source for promising hypothesis about neural calculations.

## 4. Material and methods

We test our proposed neural implementation of the discussed controller using three different systems of increasing complexity. In the beginning, we model the neural network in a simple feed-forward simulation to test its ability to extract the dominant principal component of the movement. Using the feedback of a simple, analytically solvable, mechanical system, we test the network's ability to induce stable energy-efficient movement. Finally, we demonstrate that the neural network is able to control a more realistic mechanical model of a leg with two joints that provides sensory feedback.

### 4.1. Neuron models

We model spiking neural networks, in which cells are represented either by Poisson or leaky integrate-and-fire (LIF) neurons. In every time step *dt*, the probability for a Poisson neuron to fire is given by a Poisson distribution with mean ν(*t*)*dt*, where ν(*t*) represents the instantaneous firing rate. The spike train of neuron *n* is described by $S_{n}(t)=\sum_{k}\delta (t-t_{n}^{k})$, where $t_{n}^{k}$ are the spiking times and δ denotes the delta distribution.

Where not otherwise stated, differential equations describing LIF neurons are taken from Zenke et al. (2013). Constants that have been changed in comparison to their paper are given in Table 1. Each LIF neuron has an associated membrane voltage *U*_*n*_ which changes as

(35) $$\begin{array}{r} \tau^{m}\frac{dU_{n}}{dt}=\left(U^{\mathrm{rest}}-U_{n}\right)\;+\;g_{n}^{\mathrm{exc}}\left(t\right)\left(U^{\mathrm{exc}}-U_{n}\right)\\ +\;g_{n}^{\mathrm{inh}}\left(t\right)\left(U^{\mathrm{inh}}-U_{n}\right), \end{array}$$

with membrane time scale τ^*m*^ and membrane conductances *g*^*x*^. As soon as the voltage crosses the threshold ϑ^*rest*^, a spike is triggered and *U*_*n*_ is reset to *U*^*rest*^. Our model deviates in the form of the subsequent refractory period. Zenke et al. (2013) implemented the refractory period by a time-dependent spiking threshold following a neural spike. This implementation does not consider the effect of channel inactivation on the time course of the membrane voltage, and hence its primary function is to delay the next spike. We implement a refractory period by fixing the membrane voltage of the neuron to its resting level for a time period τ_*thr*_. Our approach more closely models the absolute refractory time. It introduces a delay of same time scale between two spikes and therefore has the same functional consequence. However, it saves computational power, since the membrane and synaptic dynamics do not need to be updated during the refractory period.

**Table 1:** **Parameters of the neuron models and the structure of the neural network**.

	**Feed-Forward**	**Feedback**	
		**Simple**	**Complex**
τ_*thr*_	5 ms	5 ms	5 ms
*n*_*sens*_	1	290	130
*n*_*inh*_	0	0	100
*n*_*tim*_	0	1	6
*m*_*sens*_	/	10 Hz m^−1^	9 Hz rad^−1^
*p*_*con*_	1	1	0.7
*w*_*ext*_	/	0.1	0.1
ν_*ext*_	/	3 Hz	3 Hz
τ_*del, STDP*_	0 ms	0 ms	30 ms
τ_*del, NM*_	0 ms	0 ms	200 ms

*The feedback model is subdivided according to the simple and complex mechanical system that it controls. We state only those parameters that deviate from the original implementation as described by Zenke et al. (2013)*.

The synaptic conductances of neuron *n* are updated following a spike of the upstream neurons *m* according to

(36) $$\begin{array}{lll} g_{n}\left(t\right) & =\frac{1}{2}\left(g_{n}^{\mathrm{ampa}}\left(t\right)+g_{n}^{\mathrm{nmda}}\left(t\right)\right),\; & \; \end{array}$$

where

(37) $$\begin{array}{lll} \frac{dg_{n}^{\mathrm{ampa}}}{dt} & =-\frac{g^{\mathrm{ampa}}}{\tau^{\mathrm{ampa}}}+\underset{m}{\sum }w_{\mathrm{STDP},\mathrm{mn}}S_{m}\; & \; \end{array}$$

(38) $$\begin{array}{lll} \frac{dg_{n}^{\mathrm{nmda}}}{dt} & =-\frac{g_{n}^{\mathrm{nmda}}}{\tau^{\mathrm{nmda}}}+\frac{g_{n}^{\mathrm{ampa}}}{\tau^{\mathrm{nmda}}}.\; & \; \end{array}$$

The weight of the synapse connecting neuron *m* to *n* is given by *w*_*STDP, mn*_. The time evolution of conductances differentiates between a component due to AMPA and NMDA to account for different time constants of the corresponding channels. We modified Equation (37) to align units.

### 4.2. Plasticity

We also adapt our plasticity model from Zenke et al. (2013), who described a triplet-based STDP model based on experimental observations performed by Pfister and Gerstner (2006) and Sjöström et al. (2001). Zenke et al. defined synaptic traces $z_{n}^{\mathrm{slow}}$, $z_{n}^{-}$, and $z_{n}^{+}$ of neuron *n* by

(39) $$\begin{array}{l} \frac{dz_{n}^{x}}{dt}=-\frac{z_{n}^{x}}{\tau^{x}}+S_{n}\left(t\right). \end{array}$$

with time constants τ^*slow*^, τ^−^, and τ^−^, respectively. Synaptic weights change according to

(40) $$\begin{array}{r} \frac{dw_{\mathrm{STDP},\mathrm{nm}}}{dt}=\eta \left(A^{+}z_{n}^{+}\left(t\right)z_{m}^{\mathrm{slow}}\left(t-\epsilon \right)S_{m}\left(t\right)-A^{-}z^{-}\left(t\right)S_{n}\left(t\right)\right)\\ \;+\Delta w_{\mathrm{scal},\mathrm{nm}}\left(t\right), \end{array}$$

where ϵ is a small time constant and Δ*w*_*scal, nm*_ a homeostatic weight change as described below. Zenke et al. introduced the learning rate η as conversion factor between their plasticity model and the true biological scale, which we set to unity to match model and biological scale. They additionally scaled the rate of weight change by the initial synaptic weights. Since we consider this to be an arbitrary choice, we omit the factor. Additionally, we decrease the amplitude of long term potentiation (LTP), *A*^+^, and long term depression (LTD), *A*^−^, by two orders of magnitude (cf. Table 2). Due to the high firing rates of our neural network, we found a fast convergence of weights to their final values within tens of seconds up to minutes. Such a fast convergence would be advantageous for our consideration (cf. Section 3.1.1). However, measurements by Nishimura et al. (2013) in behaving monkeys suggest that STDP *in vivo* is more likely to act on a time scale of hours, which we account for by the decrease in amplitude. Weights in our model are altered following both pre- and postsynaptic spikes and weights of sensory neurons from joint *i* are initialized to *w*_*STDP, in*, 0_.

**Table 2:** **Parameters of the synaptic plasticity model**.

	**Feed-Forward**	**Feedback**	
		**Simple**	**Complex**
*w*_*STDP*, 1, 0_	0.5	0.7	1
*w*_*STDP*, 2, 0_	0.5	0.4	1
*A*^+^	6.5d-5	6.5d-5	6.5d-5
*A*^−^	1.1d-5	1.1d-5	1.1d-5
τ_*s*_	50 s	50 s	15,000 s
τ_*rs*_	5 s	5 s	300 s
ν_*tar*_	8 Hz	30 Hz	15 Hz

*The feedback model is subdivided according to the simple and complex mechanical system that it controls. We state only those parameters that deviate from the original implementation as described by Zenke et al. (2013)*.

To introduce stability, we use synaptic scaling as homeostatic mechanism as described by Zenke et al. (2013), who adapted it from van Rossum et al. (2000). Scaling adjusts Equation (40) by

(41) $$\begin{array}{l} \Delta w_{\mathrm{scal},\mathrm{nm}}\left(t\right)=\frac{1}{\tau_{s}\nu_{\mathrm{tar}}}\left(\nu_{\mathrm{tar}}-\left(\frac{{\bar{\nu }}_{n}}{\nu_{\mathrm{tar}}^{2}}\right)\right), \end{array}$$

where τ_*s*_ is a time constant, ν_*tar*_ the target firing rate, and ${\bar{\nu }}_{n}$ the average firing rate of the postsynaptic neuron *n* as represented by the low-pass-filtered spike train to arrive at

(42) $$\begin{array}{l} \tau_{\mathrm{rs}}\frac{d{\bar{\nu }}_{n}}{dt}=-{\bar{\nu }}_{n}+S_{n}\left(t\right). \end{array}$$

### 4.3. Neuromodulation

The motoneuron pool innvervating joint *i* starts with a serotonergic concentration of [5-HT]_*i*, 0_. Upon spiking of a raphe neuron, a fixed amount of 5-HT is released into the corresponding motoneuron pool, which subsequently diminishes according to Michaelis-Menten kinetics. The resulting concentration in motoneuron pool *i* due to the corresponding neurons *n* is described by

(43) $$\begin{array}{l} \frac{d{\left[5-HT\right]}_{i}}{dt}=c_{\mathrm{ser}}\underset{n}{\sum }S_{n}\left(t\right)-\frac{v_{\max}}{\frac{k_{m}}{{\left[5-HT\right]}_{i}}+1}, \end{array}$$

as derived in Section 2.2.2. Since we use spike-based neural networks, the firing rate in Equation (8) is replaced with the spike train. The serotonin concentration increases proportionally to the firing rate. Hence, it can be approximated that each spike releases the same quantity of serotonin. We choose *v*_*max*_ according to Hentall et al. (2006) and *k*_*m*_ as the smallest value suggested by the literature (Molodtsova, 1983; Katz and Kimelberg, 1985; Verleysdonk et al., 2004; Best et al., 2010). We set *c*_ser_ appropriately to yield [5-HT] between 0.01 and 0.1µM (Hentall et al., 2006) (cf. Table 3).

**Table 3:** **Parameters of the serotonergic dynamics model**.

	**Feed-Forward**	**Feedback**	
		**Simple**	**Complex**
[5-HT]_1, 0_	17 nM	50 nM	18 nM
[5-HT]_2, 0_	17 nM	20 nM	6 nM
*c*_*ser*_	300 pM	40 pM	5 pM
*v*_*max*_	0.1$\frac{1}{s}$	0.1$\frac{1}{s}$	0.1$\frac{1}{s}$
*k*_*m*_	170 nM	170 nM	170 nM

*The feedback model is subdivided according to the simple and complex mechanical system that it controls*.

### 4.4. Neural network

As mentioned, we test our neural network in three simulations. The computational implementation of the neural network differs for each. We use a simple computational neural model for the feed-forward simulation. A more detailed second model is used as controller for the two mechanical systems in the second and third (feedback) simulations.

#### 4.4.1. Simulation 1: feed-forward

The feed-forward network receives sensory input from two sensory Poisson neurons. They fire with mean firing rates that show a sinusoidal oscillation along a dominant eigenmode ***a*** plus a small sinusoidal component from a minor eigenmode,

(44) $$\begin{array}{l} \left(\begin{array}{c} \nu_{\varphi ,1}(t)\\ \nu_{\varphi ,2}(t) \end{array}\right)=40\mathrm{Hz}[\left(\begin{array}{c} a_{1}\\ a_{2} \end{array}\right)\sin(2\mathrm{\pi t})+\left(\begin{array}{c} 0.05\\ 0.05 \end{array}\right)\sin(8\mathrm{\pi t})\\ \;+\left(\begin{array}{c} n(\sigma =0.1)\\ n(\sigma =0.1) \end{array}\right)]. \end{array}$$

The last term represents Gaussian noise with zero mean and standard deviation σ. Negative firing rates are considered as zero. These presynaptic neurons are connected via plastic synapses to a third postsynaptic Poisson neuron that fires with a rate of

(45) $$\begin{array}{l} \nu_{\mathrm{post}}=\underset{i}{\sum }w_{\mathrm{STDP},i}\nu_{\varphi ,i}. \end{array}$$

The neuromodulator system consists of two serotonergic Poisson neurons that fire according to Equation (44) and release 5-HT into two separate motoneuron pools *i*.

#### 4.4.2. Simulation 2 and 3: feedback

The feedback neural network controlling the two mechanical systems is illustrated in Figure 4. It receives input from *n*_*sens*_ Poisson neurons per joint that fire with rates proportionally related to the respective joint deflection φ_*i*_,

(46) $$\begin{array}{l} \nu_{\varphi ,i}(t)=\{\begin{array}{ll} m_{sens}\varphi_{i}(t) & if\varphi_{i}(t)>0\\ 0 & otherwise \end{array} \end{array}$$

Sensory neurons from joint *i* are randomly connected with a probability *p*_*con*_ to each of *n*_*tim*_ LIF neurons via plastic synapses. In the network controlling the complex mechanical system, the information about spikes from the sensory neurons reaches the LIF neurons with a delay of τ_*del, STDP*_. We choose the delay in agreement with experiments on reflex arcs in the human leg (Friemert et al., 2005). Additionally, the LIF neurons receive input from *n*_*inh*_ external inhibitory Poisson neurons firing with a constant rate of ν_*ext*_. The external neurons allowed us to quickly scale the resting excitability of LIF neurons. This scaling was required due to the small number of neurons in the network. The LIF neurons accumulate synaptic input and fire as described in Section 4.1, which results in adaptation of the plastic synapses as described in Section 4.2. The spike trains of the LIF neurons are low-pass-filtered according to Equation (42) with time constant τ_*f*_ and averaged over the LIF neuron pool. This instantaneous average firing rate ${\bar{\nu }}_{\mathrm{post}}$ is proportionally converted to a generalized muscle force,

(47) $$\begin{array}{l} f_{z}\left(t\right)=m_{f}{\bar{\nu }}_{\mathrm{post}}. \end{array}$$

In the mathematical controller algorithm, converged synaptic input is transformed into an output motor signal by a step function, Equation (2). The input-output function of the LIF neurons is a smoothed approximation of this signal. The neurons are silent for small synaptic input due to the firing threshold and their firing rates saturate at high input due to the refractory period. The saturation, together with velocity dependent damping of the mechanical system as described below, ensures mechanical stability since it prevents possible positive feedback loops. For intermediate input strengths, the firing rates increase with synaptic input. The low-pass-filter, Equation (42), furthermore smooths the firing rate over time.

A number of *n*_*ser*_ serotonergic Poisson neurons fire with a rate that is linearly related to the joint coordinates,

(48) $$\begin{array}{l} \nu_{\text{ser},i}(t)=\{\begin{array}{cc} b_{\text{ser}}+m_{\text{ser}}\varphi_{i}(t)\; & \text{if}\varphi_{i}(t)>-\frac{b_{\text{ser},1}}{m_{\text{ser},1}}\\ 0\; & \text{otherwise} \end{array} \end{array}$$

Resulting spikes are delayed by τ_*del, NM*_ in the complex model. Our proposed serotonergic motor feedback loop has not been described previously and the signal delay is hence not experimentally determined. It can be assumed that the function of the network improves with shorter delays. As a safe estimation, we choose a relatively long delay which is larger than the measured delay between proprioceptive input and activity of serotonergic neurons (Springfield and Moolenaar, 1983) by one order of magnitude. Each spike increases [5-HT]_*i*_ according to Equation (43). The serotonin concentration in the motoneuron pool of the individual joints amplify the force/torque exerted on this joint proportionally, i.e., increase it by weights

(49) $$\begin{array}{l} \left(\begin{array}{c} w_{\mathrm{NM},1}\left(t\right)\\ w_{\mathrm{NM},2}\left(t\right) \end{array}\right)=c_{\mathrm{NM}}\left(\begin{array}{c} {\left[5-HT\right]}_{1}\left(t\right)\\ {\left[5-HT\right]}_{2}\left(t\right) \end{array}\right). \end{array}$$

The chosen value for *c*_*NM*_ guarantees an amplification ranging between 1 and 3 (Heckman et al., 2008).

### 4.5. Mechanical models

We test our neural feedback controller on two mechanical systems. The first consists of two masses *m* that represent the joints and are connected by a spring of stiffness *k*_1_ (cf. Figure 5A). Both masses are connected to muscles via a spring *k*_0_. The muscles exert forces on the masses according to

(50) $$\begin{array}{l} f\left(t\right)={\text{w}}_{\mathrm{NM}}\left(t\right)f_{z}\left(t\right). \end{array}$$

The deflections φ ∈ ℝ^2^ of the masses represent the joint coordinates and are measured relative to their resting positions in the absence of any force. They follow

(51) $$\begin{array}{ll} \frac{d^{2}}{dt^{2}}\varphi \left(t\right)=-\frac{d_{0}}{m}\frac{d}{dt}\varphi \left(t\right)-\left(\begin{array}{cc} \frac{k_{0}+k_{1}}{m} & -\frac{k_{1}}{m}\\ -\frac{k_{1}}{m} & \frac{k_{0}+k_{1}}{m} \end{array}\right)\varphi \left(t\right)+\frac{1}{m}\text{f}\left(t\right), & \; \end{array}$$

where *d*_0_ is a damping constant. The solution for constant muscle force is the sum of two sinusoidal oscillations with eigenvectors (1, 1) and (−1, 1). Additionally, a small damping term causes decay of minor eigenmodes. The mechanical parameters have not been specifically tuned and can be found in Table 4. Zero positions φ = 0 are defined as equilibrium positions. We excite oscillations by initially deviating the system to φ(*t* = 0*s*) = (0*m*, 0.1*m*)^*T*^. This is a linear combination of the eigenmodes. Hence, we initially excite both eigenmodes simultaneously.

**Table 4:** **Parameters of the mechanical models**.

	**Simple**		**Complex**	
τ_*f*_		100 ms		5 ms
*m*_*f*_		10 mN Hz^−1^		525 μN m Hz^−1^
*c*_*NM*_		15/μM		65/μM
*m*_*ser*_		9 Hz m^−1^		1 kHz rad−1
*b*_*ser*_		900 mHz		0 Hz
*k*_0_		8 N m−1		0.75 N m rad^−1^
*k*_1_		15 N m−1		0.75 N m rad^−1^
	*m*	500 g	*m*_0_	500 g
	*d*_0_	300 mN s m^−1^	*m*_1_	100 g
			*d*_0_	11,250 μN m s rad^−1^
			*d*_1_	11,250 μN m s rad^−1^

*Subdivided into the simple and complex model*.

The second mechanical system consists of a trunk of mass *m*_0_ connected to one rod-like thigh of mass *m*_1_ and length *l* via a rotatory hip joint (cf. Figure 6A). The shank of same mass and length is connected to the thigh via a rotatory knee joint. The joint angles define the coordinates φ∈ℝ^2^ and are measured relative to a fully extended leg. The trunk is constrained to a one-dimensional vertical jumping movement. We set its zero-position *h* = 0 to its height when both hip and knee are fully extended, i.e., φ = *0*. Gravity pulls the system down. The joints are each driven by one motor, which exerts force via a torsional spring. These springs have a torsion coefficient of *k*_0_ and *k*_1_ and an angular damping coefficient of *d*_0_ and *d*_1_ for the hip and knee joint, respectively. The equilibrium positions of the springs are defined by joint angles of $\varphi_{\text{0}}={\left(\frac{1}{6}\pi ,\frac{2}{6}\pi \right)}^{T}$. The values of these parameters, as given in Table 4, are adjusted according to an existing prototype of a legged robot at our institute. The muscles exert forces on the joints by stretching the springs. The resulting torque amounts to

(52) $$f(t)=\left(\begin{array}{c} k_{0}\\ k_{1} \end{array}\right)(w_{NM}(t)f_{z}(t)-\varphi (t)+\varphi_{0})-\left(\begin{array}{c} d_{0}\\ d_{1} \end{array}\right)\frac{d}{dt}\varphi (t).$$

After touch down, contact with the ground is modeled by forces acting on the contact point of the foot via a compliant ground model as described by Azad and Featherstone (2010) and implemented in *Spatial_v2* (Featherstone, 2012).

We define the generalized velocity $\text{v}={\left(\frac{d}{dt}h,\frac{d}{dt}\varphi \right)}^{T}$. Then, the movement can be described by

(53) $$\begin{array}{l} \text{M}\left(\varphi \right)\frac{d}{dt}\text{v}+\text{p}\left(\varphi ,\text{v}\right)=\text{f}+\underset{k}{\sum }{\text{J}}_{\text{k}}{\left(h,\varphi \right)}^{T}{\text{F}}_{\text{k}}. \end{array}$$

The inertia matrix is denoted by *M* ∈ ℝ^3 × 3^ and the Coriolis, centrifugal and gravity forces are summarized by ***p***. The ground contact wrench ***F***_***k***_ is mapped to the generalized forces by the transposed Jacobian matrix ***J***_***k***_. To initiate the movement, we drop the leg from *h* = 0m, while the joints are at φ_*0*_.

### 4.6. Parameters of the numerical simulations

We simulate our network using *Matlab* and *Simulink*. Differential equations are integrated using simple Euler integration with time steps of *dt* = 10-3 s in the feed-forward simulation and 10-4 s in the simulation comprising the simple mechanical model. To speed up the complex model, we use *dt* = 10-4 s only for the plasticity model and the LIF neurons, 10-3 s for the serotonergic dynamics, and variable time steps for the mechanical model.

### 4.7. Analysis of simulations

#### 4.7.1. Simulation 1: feed-forward

We run the feed-forward model for ten different ratios $\frac{a_{1}}{a_{2}}\in \left[0.05,0.95\right]$. Each run takes 60,000 s of simulated time. Synaptic weights are initiated as described in Table 2 and recorded each 1s during the whole simulation, serotonin concentrations are initiated as described in Table 3 and monitored in each time step during the first 500 s. During the last 50 s of the recordings, we average the ratio both of serotonergic amplification weights, $\frac{w_{\mathrm{NM},1}}{w_{\mathrm{NM},2}}$, and of the synaptic weights, $\frac{w_{\mathrm{STDP},1}}{w_{\mathrm{STDP},2}}$, for each ratio $\frac{a_{1}}{a_{2}}$ separately. Standard deviations of these values represent the fluctuation of weight ratios during the 50 s. We fit the converged ratios $\frac{w_{\mathrm{NM},1}}{w_{\mathrm{NM},2}}$ and $\frac{w_{\mathrm{STDP},1}}{w_{\mathrm{STDP},2}}$ vs. $\frac{a_{1}}{a_{2}}$ using a weighted linear least-square fit.

To verify robustness of our network against noise as stated in Equation (44), we vary the standard deviation, σ, in 10 logarithmically spaced steps between 10-2 and 100. The trial for each noise level is analyzed as described in the last paragraph. We plot the slope and intercept of the linear fit as a function of σ.

In nine additional simulations with random initial weights, we test the robustness against changes in the initial conditions. Therefore, we allocate a random weight in the interval [0.1, 1.0] to *w*_*STDP*, 1_ and *w*_*NM*, 1_ and another random weight to *w*_*STDP*, 2_ and *w*_*NM*, 2_ in each trial. We run the simulations as described above and calculate a linear fit for each of these runs. The average and standard deviation of the slope and intercept are derived over all of these nine trials plus the initial simulation.

#### 4.7.2. Simulation 2: feedback, simple mechanical model

The simulation including the simple mechanical model runs for 10,000 s of simulated time. Synaptic and neuromodulatory weights are recorded each 0.1 s only, which is due to computational restrictions. We average the weight ratios over the last 50 s of simulated time. Their respective initial values are stated in Tables 2, 3.

In nine additional trials, we verify the reliability of the results against changing initial conditions. For this test, we choose random initial concentrations [5-HT]_*i*, 0_ ∈ [6nM, 60nM] and random initial synaptic weights *w*_*STDP, i*, 0_ ∈ [0.8, 1.2] for each run. We average the ratios of synaptic and neuromodulatory weights over the last 50s of all ten trials.

#### 4.7.3. Simulation 3: feedback, complex mechanical model

We run the simulation of the neural feedback network that controls the mechanical leg for 10,000s of simulated time. Synaptic and neuromodulatory weights are recorded each 0.25 s. Their respective initial values are given in Tables 2, 3. Joint coordinates and the height of the leg are recorded for the last 70,000 time steps and for all numerical time steps during the first 50 s. Sampling of more data was not possible due to memory restrictions. We average the weight ratios during the last 50 s.

We would like to verify that the converged weight ratios maximize the energy efficiency of the movement. Therefore, we record the Euclidean vector norm of the converged synaptic and neuromodulatory weight vector of the first trial. We subsequently implement the other simulations with weight vectors that are constant over time and share the vector norm with the converged network. We vary the ratio $\frac{w_{\mathrm{STDP},1}}{w_{\mathrm{STDP},2}}=\frac{w_{\mathrm{NM},1}}{w_{\mathrm{NM},2}}$ between 0.05 and 1.5 in steps of 0.03 and run each simulation for 20 s of simulated time. In each trial, we average the jump height over the last 20 jumps.

The jump height as function of the weight ratio is taken as a measure for the energy efficiency of the neural control. At the peak of the jumping movement, the energy within the system is given by the potential energy. Since most of the mass of the system is confined to the trunk, the potential energy is approximately linearly related to the jump height. In the original bang-bang controller, switching of the bang-bang controller leads to a force *f*_*i*_ = *w*_*i*_ĉ_*f*_ in joint *i* (Equation 3). The energy inserted into the system in each jump is thus given by

(54) $$\begin{array}{l} E=\frac{1}{2}{ĉ}_{f}^{2}\left(\frac{w_{1}^{2}}{k_{0}}+\frac{w_{2}^{2}}{k_{1}}\right). \end{array}$$

We choose equal spring constants *k*_0_ = *k*_1_ for the joints. Hence, the Euclidean vector norm of the weight vector determines the energy inserted into the system. Since we fix the vector norm for each trial, the energy inserted into the system is constant. We define energy efficiency as energy within the system divided by the energy that we insert. According to our argumentation, jump height can thus be assumed to represent this quantity.

In addition, we once again perform nine trials with randomly initialized weights. The range of initial weights is the same as for the simple mechanical system described in the last section. We average the weight ratio over the last 50 s of these nine trials plus the trial described in the beginning of the present section.

To show if our neural network extracts the dominant principle component of their sensory input, we use the same ten trials with different initial conditions. As described above, we record the joint coordinates for each time step during the first 50 s of the runs. Using the Matlab-function *pca*, we extract the dominant principle component of the joint coordinates for each trial.

In a final simulation, we elucidate how the network size influences fluctuations in the jump height of the leg. We therefore increase the number of sensory input neurons to the LIF neurons by a factor of 3 and the number of LIF neurons by 2. We decrease the initial synaptic weights and the amplification factor of neuromodulation, *c*_*NM*_, by the same respective factors and run the simulation for 50 s. During the last 20 s, we calculate the standard deviation of the jump height over all jumps as a measure for the fluctuation level. Using a one-sided *F*-test, we compare this value to the standard deviation obtained for the initial simulation. The *p*-value indicates whether increasing the network size decreases fluctuations in the jumping trajectories. We use three approaches to test the assumptions underlying an *F*-test: First, we visually inspect a plot of jump height vs. time for obvious outliers for the small and large network individually. Second, we test for a significant correlation between jump height and time. Finally, we test either sample for normality using a Lillie test.

## Author contributions

AA, DL developed the hypothesis that the mathematical controller may be implemented in the nervous system. PS researched the literature. PS derived the hypothesis about the proposed serotonergic modulation. DL supported PS to find a control theoretically thorough biological implementation of DL control approach. AA, PS, DL designed the simulations. AA supervised the project. DL, PS implemented the simulations. PS ran the simulations and analyzed the results. AA, PS composed the paper. DL critically revised the paper.

### Conflict of interest statement

The authors declare that the research was conducted in the absence of any commercial or financial relationships that could be construed as a potential conflict of interest.

## Supplementary material

Video 1 **An illustration of the controller behavior**. We show here the controlled movement of our complex mechanical system, i.e., a trunk with an attached leg. An included graph shows how the serotonin concentration within the motoneuron pools of muscles innervating joint 1 (hip) and 2 (knee) converge with time. We created the animation using *Showmotion*, which is a part of *Spatial_v2* Featherstone (2012).

Presentation 1 **Proof of the convergence of our simplified version of Oja's rule, Equation (5)**.
