# Supplementary material for: Neuromodulation and Synaptic Plasticity for the Control of Fast Periodic Movement: Energy Efficiency in Coupled Compliant Joints via PCA
Source: Front Neurorobot. 2016 Mar 8;10:2. doi: 10.3389/fnbot.2016.00002 (PMC4782012; doi:10.3389/fnbot.2016.00002)
Supplement: Presentation 1 — Proof of the convergence of our simplified version of Oja's rule, Equation (5). [file Presentation1.pdf]

Supplemental Data: Neuromodulation and Synaptic Plasticity for the Control of Fast Periodic Movement: Energy Efficiency in Coupled Compliant Joints via PCA

Philipp Stratmann, Dominic Lakatos, Alin Albu-Schäffer
*Correspondence:
Philipp Stratmann
Philipp.Stratmann@dlr.de

P1 PROOF OF THE CONVERGENCE OF OUR SIMPLIFIED VERSION OF OJA'S RULE

In the following, we want to show that the update rule, eq. (5) in the article,

$$\frac{\mathrm{d}}{\mathrm{dt}}\boldsymbol{w}(t) = c_w\boldsymbol{\varphi} - \frac{1}{\tau_{eff}}\boldsymbol{w},$$

leads to a weight vector that is aligned with the result of Oja's rule, eq. (4) in the article, despite having a different magnitude. We claim that this is true as long as the weights and sensory signals are positive. We consider the case of two joints, a higher-dimensional consideration of Oja's rule (but not its simplified form) has been performed by Miller and MacKay (1994). The controller assumes that the dynamics of the oscillating mechanical system can be linearized and described by a linear second order system (Lakatos et al., 2013),

$$\ddot{\boldsymbol{\varphi}} + \boldsymbol{B}\boldsymbol{\varphi} = 0. \tag{1}$$

Using orthogonal eigenmodes $\boldsymbol{a}_k$, its trajectories are given by

$$\boldsymbol{\varphi}(t) = \sum_k \boldsymbol{a}_k \sin(\omega_k t + \phi_k) \tag{2}$$

and the dominant principal component corresponds to the dominant eigenmode $\boldsymbol{a}_1$ (Feeny and Kappagantu, 1998).

P1.1 Finding Fixed Points

We are only interested in the relative magnitude of weights corresponding to joints i, j and hence define

$$w_{i/j} := \frac{w_i}{w_j} \tag{3}$$

$$\varphi_{i/j} := \frac{\varphi_i}{\varphi_j} \tag{4}$$

to rearrange Oja's rule, eq. (4) in the article, to

$$\frac{\mathrm{d}}{\mathrm{dt}} w_{i/j} = \gamma(w_{i/j}\varphi_i + \varphi_j)\varphi_j \left[\varphi_{i/j} - w_{i/j}\right] \tag{5}$$

and the simplified update rule, eq. (5) in the article, to

$$\frac{\mathrm{d}}{\mathrm{dt}} w_{i/j} = c_w \frac{\varphi_j}{w_j} \left[\varphi_{i/j} - w_{i/j}\right] \tag{6}$$

The relative magnitude of weights is a fixed point when $\frac{d}{dt}(w_{i/j}) = 0$. For $c_w,\ w_i,\ w_j,\ \varphi_i,\ \varphi_j > 0$, both derivations imply the same fixed point

$$\frac{w_i}{w_j} = \frac{\varphi_i}{\varphi_j} = const. \tag{7}$$

This term is constant when all summands except for one have decayed in eq. (2).

P1.2 Stability

In order to prove stability, we linearize eq. (5) and eq. (6) around the fixed points. For Oja's rule we find

$$\frac{\mathrm{d}}{\mathrm{d}w_{i/j}}\left(\frac{\mathrm{d}}{\mathrm{dt}} w_{i/j}\right) = \gamma\varphi_i\varphi_j \left[\varphi_{i/j} - w_{i/j}\right] - \gamma(w_{i/j}\varphi_i + \varphi_j)\varphi_j \tag{8}$$

$$= -\gamma(\varphi_i^2 + \varphi_j^2) < 0. \tag{9}$$

For the simplified rule we find

$$\frac{\mathrm{d}}{\mathrm{d}w_{i/j}}\left(\frac{\mathrm{d}}{\mathrm{dt}} w_{i/j}\right) = c_w\varphi_j \left[\varphi_{i/j} - w_{i/j}\right] \frac{d(\frac{1}{w_j})}{dw_{i/j}} - c_w \frac{\varphi_j}{w_j} \tag{10}$$

$$= c_w\varphi_j \left[\varphi_{i/j} - w_{i/j}\right] \frac{1}{w_{i/j}} - c_w \frac{\varphi_j}{w_j} \tag{11}$$

$$= -c_w \frac{\varphi_j}{w_j}, \tag{12}$$

which is < 0 as long as weights and sensory signals are strictly positive. Therefore, both update rules converge towards the same relative weights, i.e. the weight vectors subject to either plasticity rule align.

REFERENCES

Feeny, B. and Kappagantu, R. (1998). On the physical interpretation of proper orthogonal modes in vibrations. *Journal of Sound and Vibration* 211, 607 – 616

Lakatos, D., Gorner, M., Petit, F., Dietrich, A., and Albu-Schäffer, A. (2013). A modally adaptive control for multi-contact cyclic motions in compliantly actuated robotic systems. In *Intelligent Robots and Systems (IROS), 2013 IEEE/RSJ International Conference on*. 5388–5395

Miller, K. D. and MacKay, D. J. C. (1994). The role of constraints in hebbian learning. *Neural Computation* 6, 100–126
